# Nocturnal eating disturbs phosphorus excretion in young subjects: a randomized crossover trial

**DOI:** 10.1186/s12937-015-0096-y

**Published:** 2015-10-08

**Authors:** Masae Sakuma, Saaya Noda, Yuuka Morimoto, Akitsu Suzuki, Kanaho Nishino, Sakiko Ando, Minako Umeda, Makoto Ishikawa, Hidekazu Arai

**Affiliations:** 1 Laboratory of Clinical Nutrition and Management, Graduate School of Nutritional and Environmental Sciences, The University of Shizuoka, 52-1 Yada, Suruga-ku, Shizuoka, 422-8526 Japan; 2 School of Nursing Sciences, The University of Shizuoka, Shizuoka, Japan

**Keywords:** Hyperphosphatemia, Nocturnal eating, Parathyroid hormone, Fibroblast growth factor 23, Free fatty acid

## Abstract

**Background:**

Nocturnal eating have recently increased. Serum phosphorus levels and regulators of phosphorus have circadian variations, so it is suggested that the timing of eating may be important in controlling serum phosphorus levels. However, there have been no reports on the effects of nocturnal eating on phosphorus metabolism.

The objective was to evaluate the effects of nocturnal eating on phosphorus metabolism.

**Methods:**

Fourteen healthy men participated in two experimental protocols with differing dinner times. The design of this study was a crossover study. The subjects were served test meals three times (breakfast; 07:30 h, lunch; 12:30 h, dinner; 17:30 or 22:30 h) a day. Blood and urine samples were collected to assess diurnal variation until the following morning.

**Results:**

The following morning, fasting serum phosphorus levels in the late dinner group were markedly higher than those in the early dinner group (*p* < 0.001), although serum calcium levels were maintained at approximately constant levels throughout the day in both groups. Fluctuations in urinary calcium excretion were synchronized with the timing of dinner eating, however, fluctuations in urinary phosphorus excretion were not synchronized. Urinary phosphorus excretions at night were inhibited in the late dinner group. In the late dinner group, intact parathyroid hormone levels didn’t decrease, and they were significantly higher in this group compared with the early dinner group at 20:00 h (*p* = 0.004). The following morning, fasting serum fibroblast growth factor 23 levels in the late dinner group had not changed, but those in the early dinner group were significantly increased (*p* = 0.003). Serum free fatty acid levels before dinner were significantly higher in the late dinner group compared with the early dinner group.

**Conclusions:**

Our results indicate that nocturnal eating inhibits phosphorus excretion. It is suggested that nocturnal eating should be abstained from to manage serum phosphorus levels to within an adequate range.

## Background

Phosphorus is essential for multiple and diverse biological functions, including cellular signal transduction, mineral metabolism, and energy exchange [1]. Serum phosphorus levels are maintained within a narrow range (2.5 to 4.5 mg/dL) through a complex interplay between intestinal absorption and renal excretion of phosphorus [1–3]. The progressive deterioration of renal function in chronic kidney disease leads to the retention of many substances, including phosphorus and increasing serum phosphorus levels [4]. Elevated serum phosphorus levels induces vascular calcification, arterial sclerosis and cardiovascular diseases [5–7] and has been associated with mortality in dialysis patients [8, 9]. In addition, high phosphate intake may produce detrimental health effects in the general public. There is evidence suggesting that high phosphorus intake and hyperphosphatemia may contribute to cardiovascular events among individuals with normal renal function [10–12]. Previous studies have also indicated that a high dietary phosphorus intake increases serum phosphorus levels and impairs endothelial function in healthy individuals [13, 14]. Therefore, it is recommended that not only patients with renal failure but also healthy individuals should manage their serum phosphorus levels within the appropriate range.

Nocturnal eating have recently increased due to shift work or cluttered lifestyle. According to the national health and nutrition survey in Japan, 2008, proportion of those who consume the dinner after 21:00 in over 15 years of age was 11.7 % and was increasing yearly [15]. Several studies have found a detrimental effect of night eating on a number of metabolic and cardiovascular parameters [16, 17], and rotating shift workers exhibited increased risk markers of metabolic syndrome and inflammation [18]. There has been growing evidence that when we eat is equally important to health as what we eat. Recently, the effectiveness of pharmacological and dietetic therapies appropriate to circadian rhythm was demonstrated [19, 20]. Previous studies reported that there are circadian variations in serum phosphorus levels that they are lowest in the morning and highest in the middle of the night [21], and also regulators of phosphorus such as PTH and FGF23 have circadian rhythm [21–23]. Therefore, it is suggested that the timing of eating may be important in controlling serum phosphorus levels. However, there have been no reports on the effects of nocturnal eating on phosphorus metabolism.

The purpose of this study was to evaluate the effects of the timing of eating, especially nocturnal eating, on phosphorus metabolism.

## Methods

### Subjects

Fourteen healthy men (Body mass index (BMI) 18.5-25.0 kg/m^2^) were recruited in this study. Exclusion criteria included renal dysfunction, apparent health problems and medication. Renal function and other apparent health problems were checked by a blood test. Medication was issued verbal confirmation. The study was performed after obtaining written informed consent from all of the subjects, and was approved by the Ethics Committee of the University of Shizuoka. The protocol conformed to the Helsinki Declaration.

### Study protocol

**Fig. 1:**
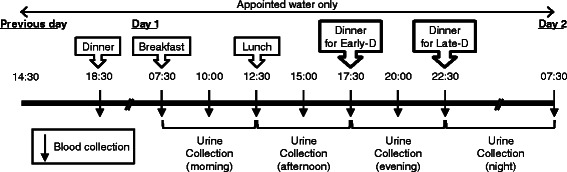
Schema of this study

Subjects participated in two experimental protocols with differing dinner times (Early dinner: Early-D and Late dinner: Late-D). The design of this experiment was a crossover study. The experiment was conducted so that each test day was separated by a washout period of at least 7 days. Outline of this study is shown in Fig. 1. The dinner time of Early-D and Late-D were 17:30 h and 22:30 h, respectively. Before each study day, all subjects were asked to avoid heavy exercise and intake of alcohol, and they were asked to abstain from foods and beverages other than appointed phosphorus free water after 14:30 h. Prescribed foods were served at 18:30 h. After an overnight fast, subjects visited the testing laboratory at 07:15 h and were asked to void. Fasting venous blood samples were collected at 07:30 h. Blood samples were collected immediately before (fasting; 07:30 h) and at 10:00, 12:30, 15:00, 17:30, 20:00, 22:30 h and next morning at 07:30 h. Subjects were served three test meals (breakfast; 07:30 h, lunch; 12:30 h and dinner; 17:30 h (Early-D) or 22:30 h (Late-D)) a day, and they consumed test meals after blood collection. All subjects drank the appointed phosphorus free water, 100 ml/h, during the experimental period. During the experimental period, subjects were asked to abstain from foods and beverages other than test meals and appointed phosphorus free water. Urine samples were collected four times over the twenty four hours: between 07:30 and 12:30 h (morning); 12:30 and 17:30 h (afternoon); 17:30 and 22:30 h (evening); and 22:30 and 07:30 h, the following morning (night). Subjects were instructed to go to bed by 24:00. We confirmed verbally that it was not different in the sleep of time between Early-D and Late-D.

### Test meals

The same constitution test meal was used for the dinner before the test day, and for breakfast, lunch and dinner on the test day (total of 4 times). The test meal consisted of 300 g steamed rice, 55 g boiled egg, 35 g ham, 150 g milk, and 2 g rice seasoning (666 kcal, 24.2 g protein, 13.8 g fat, 111.8 g carbohydrate, 400 mg phosphorus, 215 mg calcium, and 870 mg sodium). Subjects were asked to consume the test meal within 20 min. Test meal components were analyzed by the Japan food research laboratories foundation (Tokyo, Japan).

### Blood and urine analysis methods and anthropometric measurements

Serum samples were separated and stored at −30 °C until analyses of serum phosphorus, calcium, creatinine, intact parathyroid hormone (iPTH) and free fatty acid (FFA) levels were performed. Urine samples were used for analysis of phosphorus, calcium, and creatinine levels. The analysis of serum and urine samples was conducted by the blood analysis company, SRL Inc (Tokyo, Japan). Serum samples at 07:30 h and the following 07:30 h were used for analysis of serum fibroblast growth factor 23 (FGF23) levels. FGF23 can be processed by a protease to release a small C-terminal peptide which may complicate the evaluation of intact FGF23 (iFGF23) [24]. Burnett SM et al. found that iFGF23 reflects the physiological response more precisely than C-terminal FGF23 [25]. Therefore, we used an iFGF23 kit for this investigation. Anthropometric measurements were determined using a bioelectrical impedance analysis method (TANITA-RBF-215; TANITA Corporation, Tokyo, Japan). Height was measured with a stadiometer.

### Calculating formulas

We calculated the creatinine clearance (CCr), filtered phosphorus load (F-Pi), tubular threshold for phosphorus (Tp/CCr), urinary phosphorus excretion per hour (U-Pi/h) and renal fractional phosphorus excretion (FE-Pi) using the following formulas [26]. (U denotes urine, S denotes serum, P denotes phosphorus)

$$ \mathrm{C}\mathrm{C}\mathrm{r}=\mathrm{U}\hbox{-} \mathrm{volume}\times \mathrm{U}\hbox{-} \mathrm{C}\mathrm{r}\mathrm{e}\;/\;\mathrm{S}\hbox{-} \mathrm{C}\mathrm{r}\mathrm{e}\times \min $$

$$ \mathrm{F}\hbox{-} \mathrm{Pi}=\mathrm{C}\mathrm{C}\mathrm{r}\times \mathrm{S}\hbox{-} \mathrm{Pi} $$

$$ \mathrm{T}\mathrm{p}/\mathrm{C}\mathrm{C}\mathrm{r}=\mathrm{S}\hbox{-} \mathrm{Pi}\kern0.28em \hbox{-} \kern0.28em \left(\mathrm{U}\hbox{-} \mathrm{Pi}\times \mathrm{S}\hbox{-} \mathrm{C}\mathrm{r}\mathrm{e}/\mathrm{U}\hbox{-} \mathrm{C}\mathrm{r}\mathrm{e}\right) $$

$$ \mathrm{U}\hbox{-} \mathrm{Pi}/\mathrm{h}=\mathrm{U}\hbox{-} \mathrm{Pi}/\mathrm{h}\mathrm{our} $$

$$ \mathrm{F}\mathrm{E}\hbox{-} \mathrm{Pi}=\left(\mathrm{U}\hbox{-} \mathrm{Pi}\times \mathrm{S}\hbox{-} \mathrm{C}\mathrm{r}\mathrm{e}/\mathrm{U}\hbox{-} \mathrm{C}\mathrm{r}\mathrm{e}\times \mathrm{S}\hbox{-} \mathrm{Pi}\right)\times 100 $$

We also calculated the filtered calcium load (F-Ca), tubular threshold for calcium (TCa/CCr), urinary calcium excretion per hour (U-Ca/h) and renal fractional calcium excretion (FE-Ca) using the following formulas.

$$ \mathrm{F}\hbox{-} \mathrm{C}\mathrm{a}=\mathrm{C}\mathrm{C}\mathrm{r}\times \mathrm{S}\hbox{-} \mathrm{C}\mathrm{a} $$

$$ \mathrm{T}\mathrm{C}\mathrm{a}/\mathrm{C}\mathrm{C}\mathrm{r}=\mathrm{S}\hbox{-} \mathrm{C}\mathrm{a}\kern0.28em \hbox{-} \kern0.28em \left(\mathrm{U}\hbox{-} \mathrm{C}\mathrm{a}\times \mathrm{S}\hbox{-} \mathrm{C}\mathrm{r}\mathrm{e}/\mathrm{U}\hbox{-} \mathrm{C}\mathrm{r}\mathrm{e}\right) $$

$$ \mathrm{U}\hbox{-} \mathrm{C}\mathrm{a}/\mathrm{h}=\mathrm{U}\hbox{-} \mathrm{C}\mathrm{a}\;/\;\mathrm{hour} $$

$$ \mathrm{F}\mathrm{E}\hbox{-} \mathrm{C}\mathrm{a}=\left(\mathrm{U}\hbox{-} \mathrm{C}\mathrm{a}\times \mathrm{S}\hbox{-} \mathrm{C}\mathrm{r}\mathrm{e}/\mathrm{U}\hbox{-} \mathrm{C}\mathrm{r}\mathrm{e}\times \mathrm{S}\hbox{-} \mathrm{C}\mathrm{a}\right)\times 100 $$

### Statistical analysis

Data are shown as mean ± SD, and a *P* value less than 0.05 was regarded as significant. The Shapiro-Wilk statistic was performed for testing normality. Parametric analysis was used for normal distribution data, and non-parametric analysis was used for non-normal distribution data. Differences between Early-D and Late-D were calculated using the Student’s *t*-test for paired comparisons or Wilcoxon signed-rank test. Differences from baseline were calculated using a repeated measure ANOVA or Friedman's Test with Bonferroni post hoc test. All of the statistical analyses were performed using the Statistical Package of Social Science (SPSS for Windows, version 19.0, SPSS, Chicago, IL).

## Results

### Characteristics of the subjects

The clinical and biological characteristics of the subjects are shown in Table 1. The mean values ± SD of age and BMI were 22.8 ± 1.8 years and 22.5 ± 2.2 kg/m^2^, respectively. Their glucose metabolism, hepatic function and renal function were normal.

**Table 1:** Characteristics of the subjects

Characteristic		Mean ± SD
Age	(year)	22.8 ± 1.8
Height	(cm)	170.0 ± 5.8
Body weight	(kg)	64.9 ± 8.7
Body fat percentage	(%)	18.4 ± 3.6
BMI	(kg/m^2^)	22.5 ± 2.2
Triglyceride	(mg/dL)	69.7 ± 22.0
LDL-cho	(mg/dL)	81.8 ± 15.7
HDL-cho	(mg/dL)	60.0 ± 11.4
HbA1c	(%)	5.3 ± 0.3
Total protein	(g/dL)	7.3 ± 0.4
Albumin	(g/dL)	4.8 ± 0.3
UN	(mg/dL)	11.6 ± 2.4
Creatinine	(mg/dL)	0.9 ± 0.1
Na	(mEq/L)	140.2 ± 1.2
K	(mEq/L)	4.2 ± 0.1
Ca	(mg/dL)	9.6 ± 0.4
Pi	(mg/dL)	4.0 ± 0.5
intact PTH	(pg/dL)	39.4 ± 11.6

All values are mean ± SD; *n* = 14. *BMI*: body mass index; *LDL-cho*: low density lipoprotein-cholesterol; *HDH-cho*: high density lipoprotein-cholesterol; *HbA1c*: hemoglobin A1c; *UN*: urea nitrogen; *Na*: sodium; *K*: potassium; *Ca*: calcium; *Pi*: phosphorus; intact *PTH*: intact parathyroid hormone

### Serum phosphorus, calcium and iPTH levels

**Fig. 2:**
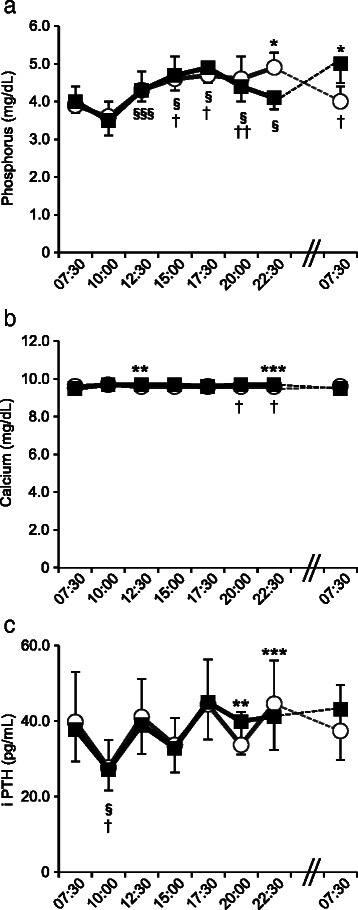
Change in serum phosphorus, calcium and iPTH levels. Open circle: Early-D; closed square: Late-D. **(a)** serum phosphorus levels, (**b**) serum calcium levels, (**c**) serum iPTH levels. Differences between Early-D and Late-D were calculated using the Student’s *t*-test for paired comparisons or Wilcoxon signed-rank test. Differences from baseline were calculated using a repeated measure ANOVA or Friedman's Test with Bonferroni post hoc test. Early-D: early dinner; Late-D: late dinner; iPTH: intact parathyroid hormone. *P < 0.001, **P < 0.01, ***P < 0.05 Early-D versus Late-D. §*P* < 0.001, §§§*P* < 0.05 versus baseline for Early-D. †*P* < 0.001, ††*P* < 0.01 versus baseline for Late-D

The circadian variations in serum phosphorus, calcium and iPTH levels are shown in Fig. 2. In Early-D and Late-D, serum phosphorus levels slightly decreased after breakfast, and then increased over time. Serum phosphorus levels at 12:30, 15:00, 17:30, 20:00 and 22:30 h were significantly higher than baseline in Early-D (*P* = 0.037; 12:30 h, *P* < 0.001; 15:00, 17:30, 20:00 and 22:30 h). In Late-D, serum phosphorus levels at 15:00, 17:30, 20:00 h and following morning at 07:30 h were significantly higher than baseline (*P* < 0.001; 15:00, 17:30 h and following morning at 07:30 h, *P* = 0.005; 20:00 h). Serum phosphorus levels at 22:30 h in Late-D were significantly lower compared with those in Early-D (*P* < 0.001) because Late-D had not yet consumed dinner. The following morning at 07:30 h, fasting serum phosphorus levels in Late-D were markedly higher than in Early-D (Early-D; 4.0 ± 0.4 mg/dL, Late-D; 5.0 ± 0.5 mg/dL; *P* < 0.001) (Fig. 2a).

Serum calcium levels were maintained at approximately constant levels throughout the day in Early-D and Late-D (Fig. 2b).

In Early-D, iPTH levels were decreased 2.5 h after each meal (10:00, 15:00 and 20:00 h) and increased before each meal (12:30, 17:30 and 22:30 h). In Late-D, iPTH levels did not decrease at 20:00 h because of continued fasting to 22:30 h, and were significantly higher in Late-D compared with Early-D at 20:00 h (*P* = 0.004) (Fig. 2c).

### Renal phosphorus metabolic indices

**Fig. 3:**
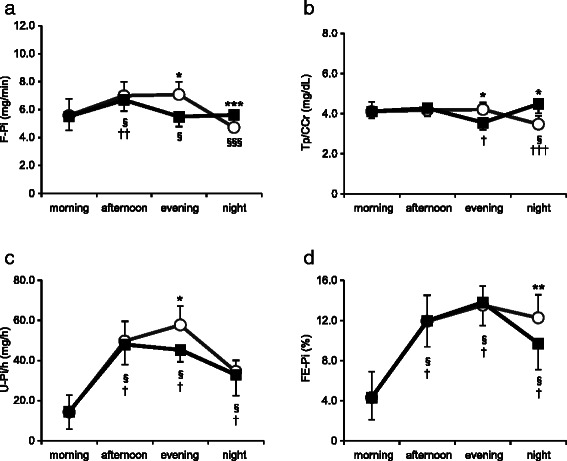
Time course of renal phosphorus metabolic indices. Open circle: Early-D; closed square: Late-D. **(a)** filtered phosphorus load (F-Pi), (**b**) tubular threshold for phosphorus (Tp/CCr), (**c**) urinary phosphorus excretion per hour (U-Pi/h), (**d**) renal fractional phosphorus excretion (FE-Pi). Differences between Early-D and Late-D were calculated using the Student’s *t*-test for paired comparisons or Wilcoxon signed-rank test. Differences from baseline were calculated using a repeated measure ANOVA or Friedman's Test with Bonferroni post hoc test. Early-D: early dinner; Late-D: late dinner. *P < 0.001, **P < 0.01, ***P < 0.05 Early-D versus Late-D. §*P* < 0.001, §§§*P* < 0.05 versus baseline for Early-D. †*P* < 0.001, ††*P* < 0.01, †††*P* < 0.05 versus baseline for Late-D

The time course of renal phosphorus metabolic indices is shown in Fig. 3. There were no differences in filtered phosphorus load (F-Pi) between the Early-D and Late-D groups at the morning and afternoon measurements, however, F-Pi at the evening measurement was significantly higher in Early-D compared with Late-D (*P* < 0.001) and F-Pi at the night measurement was significantly higher in Late-D than in Early-D (*P* = 0.019). F-Pi fluctuated in synchronization with the timing of meals (Fig. 3a). In addition, fluctuations in the tubular threshold for phosphorus (Tp/CCr) were synchronized with the timing of meals, Tp/CCr at the evening measurement was significantly higher in Early-D compared with Late-D (*P* < 0.001) and Tp/CCr at the night measurement was significantly higher in Late-D compared with Early-D (*P* < 0.001) (Fig. 3b). Urinary phosphorus excretion per hour (U-Pi/h) at the evening measurement was significantly lower in Late-D compared with Early-D (*P* < 0.001) and there was no difference in U-Pi/h at the night measurement between Early-D and Late-D (Early-D; 34.5 ± 5.6 mg/h, Late-D; 32.9 ± 10.3 mg/h; *P* = 0.149) (Fig. 3c). Renal fractional phosphorus excretion (FE-Pi) at the night measurement was also significantly lower in Late-D compared with Early-D (*P* = 0.004) (Fig. 3d). 24-h total urinary phosphorus excretion was significantly lower in Late-D than those in Early-D (Early-D; 919.3 ± 97.4 mg, Late-D; 835.0 ± 129.4 mg; *P* = 0.043).

### Renal calcium metabolic indices

**Fig. 4:**
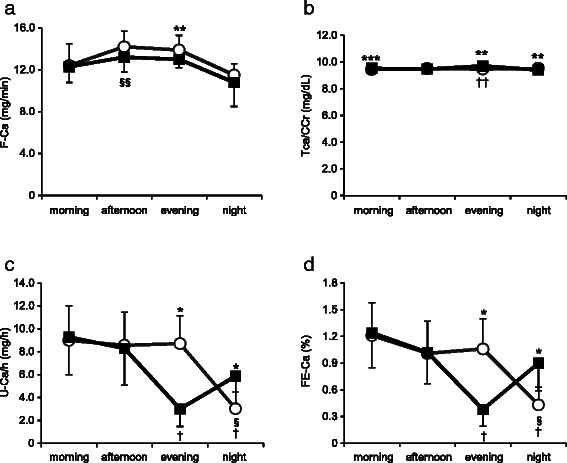
Time course of renal calcium metabolic indices. Open circle: Early-D; closed square: Late-D. (**a**) filtered calcium load (F-Ca), (**b**) tubular threshold for calcium (TCa/CCr), (**c**) urinary calcium excretion per hour (U-Ca/h), (**d**) renal fractional calcium excretion (FE-Ca). Differences between Early-D and Late-D were calculated using the Student’s *t*-test for paired comparisons or Wilcoxon signed-rank test. Differences from baseline were calculated using a repeated measure ANOVA or Friedman's Test with Bonferroni post hoc test. Early-D: early dinner; Late-D: late dinner. *P < 0.001, **P < 0.01, ***P < 0.05 Early-D versus Late-D. §*P* < 0.001, §§*P* < 0.01 versus baseline for Early-D. †*P* < 0.001, ††*P* < 0.01 versus baseline for Late-D

The time course of renal calcium metabolic indices is shown in Fig. 4. Filtered calcium load (F-Ca) slightly increased during the diurnal period. Although F-Ca at the evening measurement was significantly higher in Early-D compared with Late-D (*P* = 0.007), there was little difference between Early-D and Late-D (Fig. 4a). The tubular threshold for calcium (TCa/CCr) was maintained at approximately constant levels throughout the day in Early-D and Late-D (Fig. 4b). Urinary calcium excretion per hour (U-Ca/h) and renal fractional calcium excretion (FE-Ca) at the evening measurements were significantly higher in Early-D compared with Late-D (*P* < 0.001) and at the night measurements, they were significantly higher in Late-D compared with Early-D (*P* < 0.001). Fluctuations in U-Ca/h and FE-Ca were synchronized with the timing of meals, unlike U-Pi/h and FE-Pi (Fig. 4c, d). 24-h total urinary calcium excretion was not significantly different between in Late-D and in Early-D (Early-D; 158.6 ± 46.9 mg, Late-D; 155.7 ± 52.1 mg; *P* = 0.713).

### Serum FGF-23 levels

On day 1, there were no differences in fasting serum FGF23 levels between Early-D and Late-D (Early-D; 44.5 ± 13.9 pg/mL, Late-D; 46.7 ± 13.3 pg/mL; *P* = 0.603). There were no differences in fasting serum FGF23 levels in Late-D between day 1 and day 2 (day 1; 46.7 ± 13.3 pg/mL, day 2; 47.7 ± 14.4 pg/mL; *P* = 0.682). However, in Early-D, fasting serum FGF23 levels were significantly higher on day 2 compared with day 1 (day 1; 44.5 ± 13.9 pg/mL, day 2; 53.1 ± 14.9 pg/mL; *P* = 0.003) (Table 2).

**Table 2:** Serum FGF 23 levels

Day 1		Day 2	
Early-D	Late-D	Early-D	Late-D
44.5 ± 13.9	46.7 ± 13.3	53.1 ± 14.9*	47.7 ± 14.4

*FGF 23*: fibroblast growth factor 23; *Early-D*: early dinner; *Late-D*: late dinner

*Significantly different from the Day 1, *P* = 0.003 (student’s *t*-test for paired comparisons)

### Serum FFA levels

**Fig. 5:**
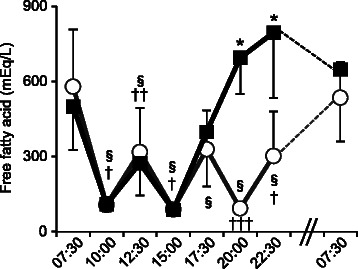
Change in serum free fatty acid levels. Open circle: Early-D; closed square: Late-D. Differences between Early-D and Late-D were calculated using the Wilcoxon signed-rank test. Differences from baseline were calculated using a Friedman's Test with Bonferroni post hoc test. Early-D: early dinner; Late-D: late dinner. *P < 0.001 Early-D versus Late-D. §*P* < 0.001 versus baseline for Early-D. †*P* < 0.001, ††*P* < 0.01, †††*P* < 0.05 versus baseline for Late-D

Serum FFA levels decreased 2.5 h after a meal and increased 5 h after a meal (breakfast, lunch and dinner). Serum FFA levels in Late-D continued rising from 17:30 to 22:30 h. Serum FFA levels at 20:00 and 22:30 h in Late-D were significantly higher than in Early-D (*P* < 0.001; 20:00 and 22:30 h). Serum FFA levels before the evening meal were significantly higher in Late-D (22:30 h; 648.2 ± 287.3 mEq/L) compared with Early-D (17:30 h; 328.9 ± 155.5 mEq/L) (*p* < 0.001) (Fig. 5).

## Discussion

In this study, we evaluated the effects of the timing of the evening meal on phosphorus metabolism. Nocturnal eating in Late-D caused an increase in serum phosphorus levels the following morning (next 07:30 h), and the serum phosphorus levels exceeded the reference levels (2.5 to 4.5 mg/dL). Serum phosphorus levels at 22:30 h was above the reference levels in Early-D. This phenomenon is thought to be derived to a circadian variation of serum phosphorus. Previous study reported that serum phosphorus levels were lowest in the morning and highest in the middle of the night, and they were greatly modified to the reference levels during a prolonged fast [21]. If the serum phosphorus levels in the morning are higher than the reference value such as Late-D in this study, there is a possibility that the serum phosphorus levels in the afternoon and the middle of the night further rises due to the diurnal variation. As a result, It might be manifested the hyperphosphatemia throughout the day. Also, considering there were 9 h between the measurements, serum phosphorus levels were estimated to be higher at midnight than levels at 07:30 h the following morning in Late-D. There are four major possible reasons for the observed serum phosphorus levels. First, the effect of PTH was considered. PTH increases urinary excretion of phosphorus by lowering renal sodium-phosphate transporter (NaPi-2a and NaPi-2c) expression [27]. In addition, PTH stimulates calcium release from bone and intestinal absorption of calcium by elevating renal production of 1, 25-OH_2_D_3_ [28]. Secretion of PTH is induced by lowering serum calcium levels [29] and diminished by increasing serum calcium levels (including through feeding) [30]. PTH circadian rhythmicity is well established in healthy individuals, and there is increasing evidence that fluctuations in PTH secretion may have an important effect in governing normal bone health, bone turnover, and bone remodeling [21, 31–34]. It is reported that serum PTH levels gradually increase from 20:00 h, and reach maximum levels between 02:00 and 04:00 h [21, 22]. In Late-D, eating dinner at 22:30 h raised serum calcium levels at the time of ordinary increasing PTH levels, consequently, PTH secretion in the middle of the night might be prevented. As a result, it is possible that serum phosphorus levels were increased the next morning in Late-D because phosphorus excretion during the middle of the night was inhibited. In this study, U-Pi/h increased after dinner in Early-D (evening), but did not increase after dinner in Late-D (night), supporting our hypothesis that nocturnal eating inhibits phosphorus excretion during the middle of the night. The fact that 24-h total urinary phosphorus excretion in Late-D was significantly lower than those in Early-D also supports our hypothesis. The possibility that low serum phosphorus level at 22:30 in Late-D increased tubular phosphorus reabsorption to approximate ideal serum phosphorus levels is thought. Alternately, U-Ca/h increased after dinner in both Early-D and Late-D, and there were no differences in serum calcium levels between Early-D and Late-D the following morning. Calcitonin and PTH are serum calcium level-regulating hormones. A previous study indicated that neither increased dietary calcium nor calcium supplements affected plasma calcitonin responses [35], therefore it would be necessary to suppress PTH secretion in the middle of the night in Late-D to keep serum calcium levels in the normal range. Calcium sensing receptors play a crucial role in detecting extracellular calcium levels and initiating the synthesis and secretion of PTH, which is suppressed when the extracellular calcium concentration is increased [36]. Serum calcium levels are maintained within a very narrow range (8.4 to 10.0 mg/dL; 2.1 to 2.5 mmol/L) compared to serum phosphorus levels (2.5 to 4.5 mg/dL; 0.8 to 1.6 mmol/L). It is thought that PTH preferentially regulated calcium rather than phosphorus in this study.

The second possible factor affecting serum phosphorus levels may be the effects of FFA. High levels of serum FFA may contribute to insulin resistance, increased hepatic glucose production [37, 38], and reduced glucose utilization [39, 40]. It was reported in a study using vascular smooth muscle cell that an insulin resistant state decreased the cellular uptake of phosphorus [41]. Serum FFA levels before dinner in Late-D (22:30 h; 648.2 ± 287.3 mEq/L) were significantly higher than those in Early-D (17:30 h; 328.9 ± 155.5 mEq/L). These results suggest that cellular uptake of phosphorus was attenuated by insulin resistance while we cannot affirm because we did not measure plasma glucose and serum insulin levels. Another study showed that an oral glucose load that induced hyperglycemia/hyperinsulinemia promoted a significant decline in serum PTH in postmenopausal women [42]. It is possible that temporary insulin resistance at the dinner time induced rapid elevation in glucose and insulin levels, and suppressed PTH secretion in Late-D.

The third reason concerns the effects of FGF23. FGF23 is a recently identified molecule involved in the control of phosphorus homeostasis. FGF23 reduces serum phosphorus levels by decreasing renal phosphorus reabsorption and intestinal phosphorus absorption [43–48]. In a previous study, serum FGF23 levels were slightly decreased up to 8 h after intake of 400 mg phosphorus. A significant increase in serum FGF23 levels were observed 8 h after intake of 1200 mg phosphorus compared with intake of 400 and 800 mg phosphorus, suggesting that it requires a relatively long time to secrete FGF23 after eating [13]. In this study, the time from dinner intake to the next morning (fasting blood collection) was 14 h in Early-D and 9 h in Late-D. Serum FGF23 levels at the next morning (Day 2) in Late-D were not elevated compared with Day 1, but those in Early-D were elevated. We suggest it took more than 9 h before FGF23 was secreted due to the intake of 400 mg phosphorus per meal.

The fourth possible factor is the effect of growth hormone (GH), which involves inhibition of renal phosphorus reabsorption and excretion as well as bone growth and enlargement of organs such as muscle, heart, liver and kidney. Secretion of GH is stimulated in the middle of the night and serum GH levels almost coincides with the highest serum phosphate and PTH levels [21]. It is possible that the secretion of GH increased before the phosphorus contained in the dinner was excreted, thus preventing phosphorus excretion in Late-D.

It is reported that the circadian rhythm of serum phosphorus levels was affected by feeding time rather than light–dark cycle [49]. In a previous observation, ingested diets containing 625 or 2300 mg phosphorus induced little or no change in morning fasting serum phosphorus levels in healthy men. In that study, dinner was consumed at 17:15 h [50]. These previous studies support our observation that it is not only the “amount of intake” but also the “timing of eating” which plays an important role in the control of serum phosphorus levels.

In conclusion, our results indicate that nocturnal eating leads to hyperphosphatemia. It is suggested that nocturnal eating should be abstained from to manage serum phosphorus levels to within an adequate range.

## Abbreviations

BMI: Body mass index
CCr: Creatinine clearance
Early-D: Early dinner
F-Ca: Filtered calcium load
FE-Ca: Renal fractional calcium excretion
FE-Pi: Renal fractional phosphorus excretion
FFA: Free fatty acid
FGF23: Fibroblast growth factor 23
F-Pi: Filtered phosphorus load
GH: Growth hormone
iPTH: Intact parathyroid hormone
Late-D: Late dinner
TCa/CCr: Tubular threshold for calcium
Tp/CCr: Tubular threshold for phosphorus
U-Ca/h: Urinary calcium excretion per hour
U-Pi/h: Urinary phosphorus excretion per hour
